# Circ0060467 sponges miR-6805 to promote hepatocellular carcinoma progression through regulating AIFM2 and GPX4 expression

**DOI:** 10.18632/aging.205460

**Published:** 2024-01-19

**Authors:** Ye-Ru Tan, Bao-Hong Jiang, Wen-Jie Feng, Zhi-Long He, Yi-Ling Jiang, Yi Xun, Xiao-Ping Wu, Yue-Hua Li, Hong-Bo Zhu

**Affiliations:** 1 Department of Medical Oncology, The First Affiliated Hospital, Hengyang Medical School, University of South China, Hengyang, Hunan Province, China; 2 Department of Pharmacy, The First Affiliated Hospital, Hengyang Medical School, University of South China, Hengyang, Hunan Province, China

**Keywords:** circRNAs, miR-6085, AIFM2, GPX4, hepatocellular cancer (HCC)

## Abstract

Background: Circular RNAs (circRNAs) represent a subset of non-coding RNAs implicated in the regulation of diverse biological processes, including tumorigenesis. However, the expression and functional implications of circ0060467 in hepatocellular carcinoma (HCC) remain elusive. In this study, we aimed to elucidate the role of circ0060467 in modulating the progression of HCC.

Methods: Differentially expressed circRNAs in HCC tissues were identified through circRNA microarray assays. Quantitative reverse transcription polymerase chain reaction (qRT-PCR) assays revealed the upregulation of circ0060467 in both HCC cell lines and tissues. Various assays were conducted to investigate the roles of circ0060467 in HCC progression. Additionally, RNA immunoprecipitation (RIP) assays and luciferase assays were carried out to assess the interactions between circ0060467, microRNA-6085 (miR-6085), apoptosis-inducing factor mitochondria-associated 2 (AIFM2), and glutathione peroxidase 4 (GPX4) in HCC.

Results: Microarray and qRT-PCR analyses demonstrated a marked elevation of circ0060467 in HCC tissues and cell lines. Knockdown of circ0060467 suppressed HCC cell proliferation. Luciferase reporter and RIP assays confirmed the binding of circ0060467, AIFM2, and GPX4 to miR-6805. Subsequent experiments revealed that circ0060467 competes with AIFM2 and GPX4, thereby inhibiting cancer cell ferroptosis by binding to miR-6085 and promoting hepatocellular carcinoma progression.

Conclusions: Collectively, circ0060467 modulates the levels of AIFM2 and GPX4, crucial regulators of tumor cell ferroptosis, by acting as a sponge for miR-6085 in HCC. Thus, circ0060467 may represent a novel diagnostic marker and therapeutic target for HCC.

## INTRODUCTION

Hepatocellular carcinoma (HCC) is a common solid tumor and a major cause of cancer-associated death worldwide. In 2020, primary liver cancer became the 6^th^ frequently diagnosed carcinoma and the 3^rd^ main cause of tumor-associated deaths globally, with about 906,000 new morbidities and 830,000 mortalities [1]. Geographically, the incidence of HCC exhibits notable disparities, predominantly concentrated in less developed regions such as Eastern Asia (54.8% of cases) and Southeastern Asia (10.8% of cases) [2–4]. Despite advancements in the treatment of HCC, the prognostic outcomes for afflicted patients remain bleak [5–7]. While numerous genes are implicated in HCC tumorigenesis, the underlying pathogenic mechanisms remain elusive [8–10]. Consequently, there exists an imperative need to comprehend these mechanisms comprehensively and identify novel molecular targets to furnish efficacious therapeutic interventions for HCC [11].

Circular RNAs (circRNAs) represent a category of non-coding RNAs ubiquitous across various species [12, 13]. Distinguished by their highly stable and evolutionarily conserved circular structures, circRNAs inherently demonstrate resilience to RNase activity. Moreover, specific miRNA-binding sites within circRNA sequences endow them with the capacity to function as miRNA sponges, thereby exerting regulatory control over gene expression. The aberrant expression of circRNAs is frequently discerned in progressive tumors, owing to their role as miRNA sponges [14, 15].

Previous research has demonstrated that circRNAs play a pivotal role in co-regulating other RNAs by functioning as competing endogenous RNAs (ceRNAs), thereby competing for microRNAs (miRNAs) [16]. Kong et al. reported that cirPLK1 functions as a miR-296-5p sponge to suppress progression of triple-negative breast cancer [17]. Similarly, CircSMARCA5 plays a role in HCC proliferation as well as metastasis by acting as a miRNA sponge for miR-181b-5p and miR-17-3p [18]. These findings collectively underscore the pivotal role of circRNAs as modulators of cancer progression through their function as miRNA sponges.

Several circRNAs have been identified as critical regulators in the early stages of HCC progression. Notably, Circ-CDYL exhibits high expression levels in early-stage HCC, where it downregulates the expression of Hepatoma-Derived Growth Factor (HDGF) and Hypoxia-Inducible Factor 1 Alpha Inhibitor (HIF1AN) by acting as a ceRNA for both miR-328-3p and miR-892a [19]. Another noteworthy circRNA, Circ-PRKCI, functions as a miRNA-545 sponge, antagonizing the E2F transcription factor 7 (E2F7) [20]. E2F7 significantly overexpressed in HCC, is associated with a poor survival outcome. Moreover, the upregulation of hsa-circ-0078710 in HCC has been closely linked to cancer cell proliferation, migration, and invasion. Functioning as a miR-31 sponge, has-circ-0078710 activates CDK2 and HDAC genes [21]. However, the expression and functional role of circ0060467 in HCC remain unclear and warrant further investigation.

Ferroptosis, a term coined by Dixon et al. in 2012 [22], denotes a form of iron-dependent regulation of cell death driven by an overload of lipid peroxides on the cell membrane. It exhibits distinct morphological and mechanistic characteristics when compared to apoptosis and other types of cell death [22, 23]. In recent years, enlightening progress has made in cancer research demonstrating that ferroptosis produces either a facilitative or inhibitory effect on cancer development, and has potential as a new target for cancer therapy. On the one hand, several cancer-related signaling pathways have been demonstrated to be closely associated with and involved in regulating the process of ferroptosis [24]. These pathways consequently influence the metabolic, immune, and drug-sensitive characteristics of tumor cells [25–27]. The distinctive metabolism of tumor cells renders them susceptible to ferroptosis, indicating heightened sensitivity to targeted therapy in specific types of cancer [28–30]. Therefore, investigating the vulnerability of HCC to ferroptosis and comprehending the specific regulatory mechanisms assumes paramount significance in the prevention and treatment of HCC.

In this study, the expression of circ0060467 was determined in 18 paired HCC tissues utilizing quantitative reverse transcription polymerase chain reaction (qRT-PCR) [18]. Circ0060467 was detected significantly upregulated in HCC tissues and cell lines. Circ0060467 is located at chr20:42338602-42345122 and derived from the MYB proto-oncogene like 2 (MYBL2) gene. Previous studies have indicated its upregulation in cervical cancer, contributing to increased paclitaxel resistance, and fostering cancer proliferation by modulating miR-665/EGFR signaling [31]. Notably, the knockdown of circ0060467 has been shown to suppress proliferation and enhance FLT3-ITD acute myeloid leukemia cell differentiation by promoting FLT3 kinase translational efficiency [32]. Here, we explored the role of circ0060467 in HCC and found that circ0060467 knockdown suppressed cell proliferation. We confirmed that circ0060467 bound to miR-6805. Notably, we identified Apoptosis-Inducing Factor Mitochondria Associated 2 (AIFM2) and Glutathione Peroxidase 4 (GPX4), pivotal regulators of tumor ferroptosis, as direct targets of miR-6805. Consequently, our results elucidate the pivotal role of circ0060467 as a miR-6805 sponge, orchestrating a ceRNA mechanism to modulate the expression of AIFM2 and GPX4. This intricate regulatory network positions circ0060467 as a potential therapeutic target for HCC.

## RESULTS

### Elevated expression of circ0060467 in HCC cell lines and tissues

**Figure 1:**
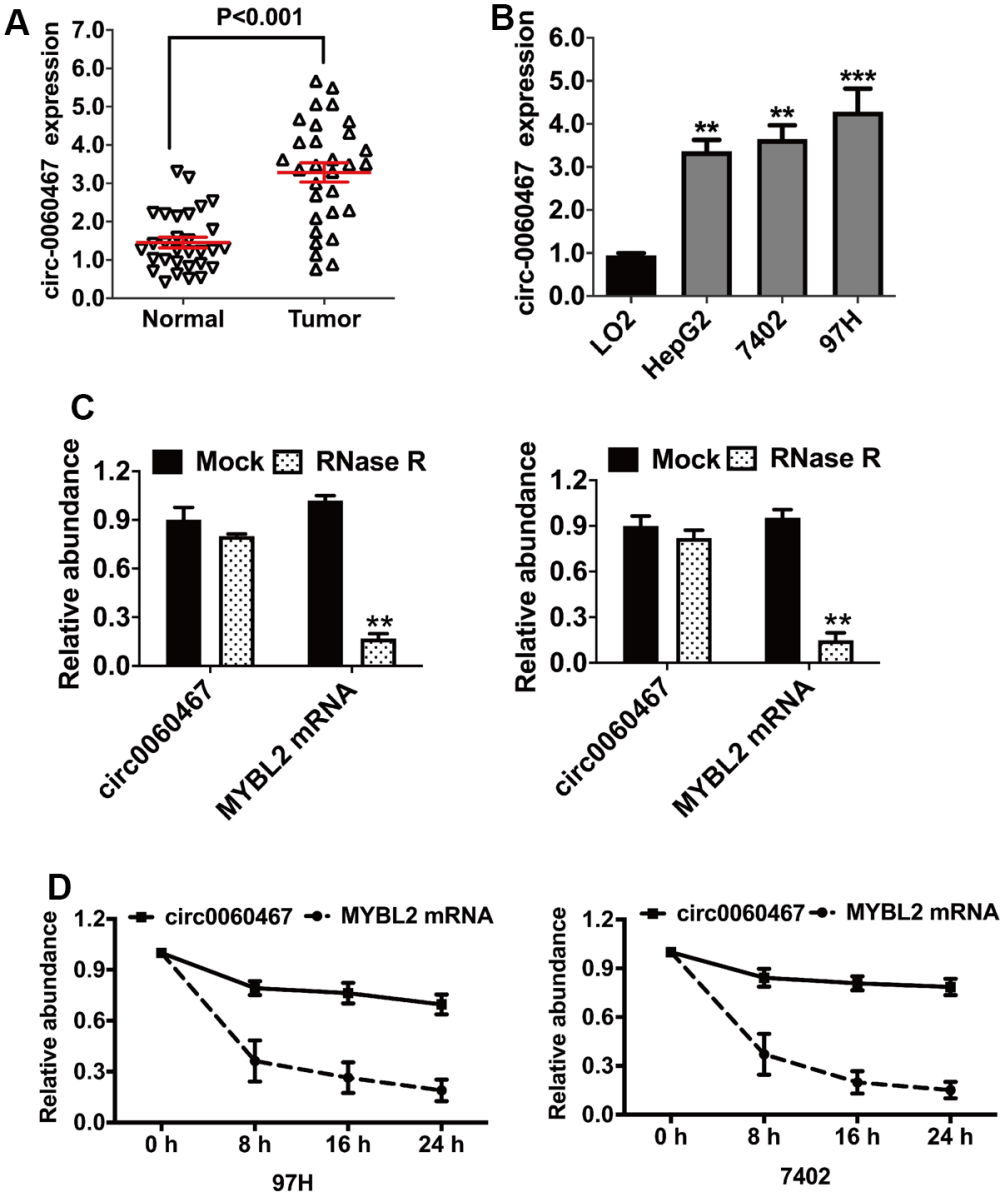
**Circular RNA hsa_circ_0060467 (circ0060467) is increased in hepatocellular carcinoma (HCC) cell lines and tissues.** (**A**) Expression level of circ0060467 in normal and HCC cell lines. (**B**) Expression level of circ0060467 in HCC tissues and matched normal tissues. (**C**) RNase R digestion experiment was used to confirm the circular structure of circ0060467 in 97H (left) and 7402 cells (right). (**D**) Stability of the circular transcript circ0060467 and linear transcript MYBL2 in 97H and 7402 cells assessed by actinomycin D assays. **p < 0.01, *** *p*<0.001.

The upregulation of circ0060467 has been observed in various cancers, where it functions as an oncogene [31, 32]. A previous investigation identified circ0060467 in 18 paired cervical cancer tissues, demonstrating its significance in cancer biology [18]. Specifically, it was revealed that circ0060467 serves as a miR-361-3p sponge, augmenting the growth and invasion of cervical tumor cells. However, the functional and mechanistic aspects of circ0060467 in HCC remain unclear. In this study, an examination of circ0060467 expression revealed its elevation in both HCC tissues and cells (Figure 1A, 1B). The circular structure of circ0060467 was confirmed by RNase R digestion experiments in 97H and 7402 cells (Figure 1C). Further, findings from the actinomycin D assay implied that the circular transcript circ0060467 in 97H and 7402 cells was highly stable, compared to its linear transcript MYBL2 (Figure 1D).

### Suppression of circ0060467 inhibits HCC tumor proliferation

**Figure 2:**
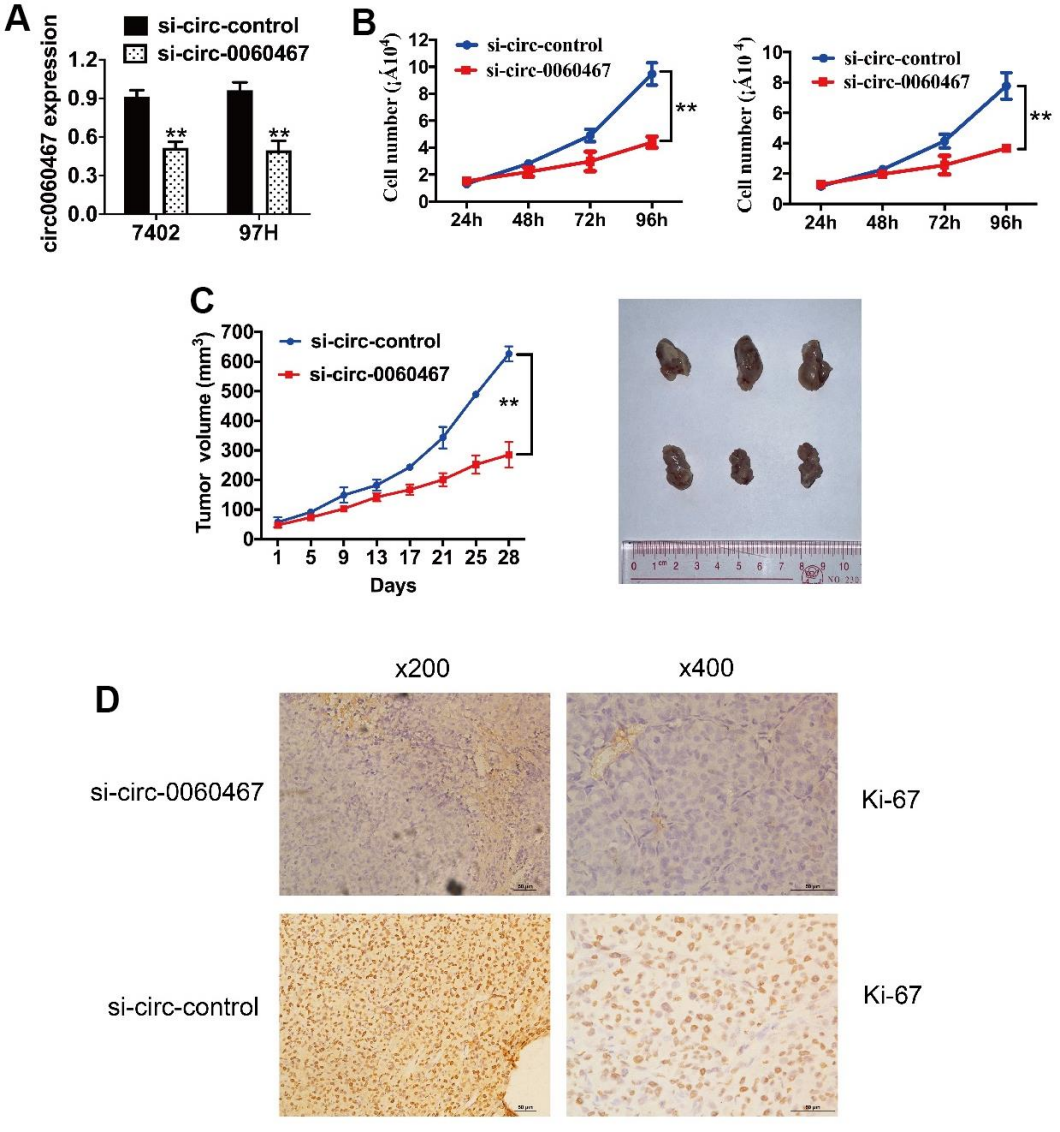
**Knockdown of circ0060467 suppresses HCC tumor growth.** (**A**) si-circ0060467 successfully knocked down circ0060467 in 7402 (left) and 97H (right) cells. (**B**) Cell Counting Kit-8 assay was performed to evaluate cell proliferation in 7402 and 97H cells. (**C**) Tumor volume was measured every 4 days for 4 weeks. (**D**) Expression status of Ki67 in hematoxylin-eosin-stained sections of harvested xenograft tumors. **p < 0.01.

To assess the roles of circ0060467 in HCC, RNA interference was used to downregulate circ0060467 levels and the successful inhibition was showed by qRT-PCR analysis (Figure 2A). Notably, the suppression of circ0060467 markedly inhibited cell growth (Figure 2B). To evaluate the impact of circ0060467 on *in vivo* tumor proliferation, a xenograft experiment was conducted. It showed that tumor proliferation was significantly suppressed with inhibition of circ0060467 (Figure 2C). Moreover, immunohistochemical staining of xenograft tumor sections indicated suppression of Ki-67 levels following the inhibition of circ0060467 (Figure 2D).

### Circ0060467 functions as an miR-6085 sponge

**Figure 3:**
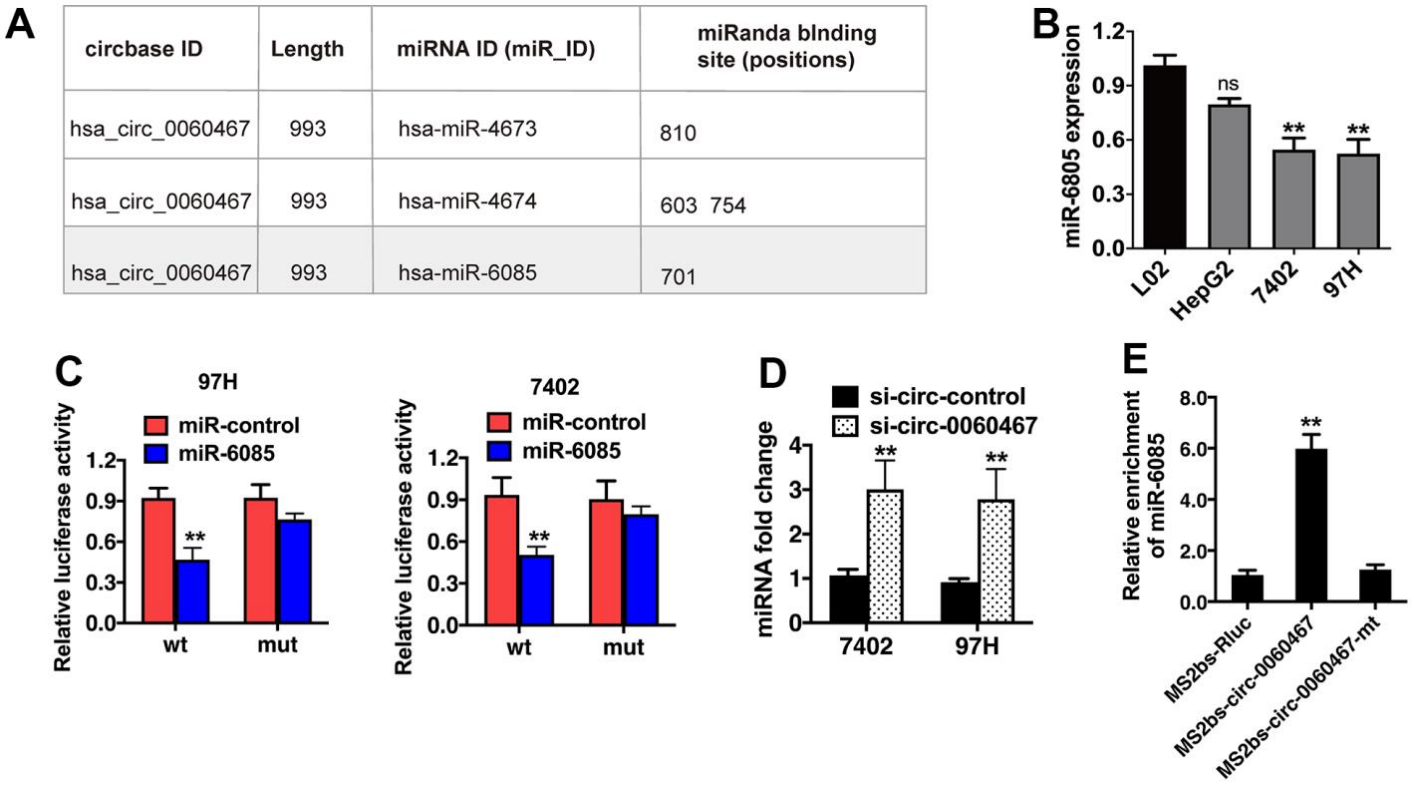
**Circ0060467 functions as a sponge for miR-6085 in hepatocellular carcinoma (HCC) cells.** (**A**) Predicted binding sites of miR-6085 within the circ0060467 sequence. (**B**) Expression level of miR-6085 in the LO2 cell line and HCC cell lines. (**C**) Relative luciferase activity of 97H and 7402 cells co-transfected with the miR-6085 mimics and the luciferase reporter vector containing the wild-type or mutant circ0060467 sequence. (**D**) miR-6085 expression in 7402 and 97h cell lines with knockdown of circ-0060467. (**E**) MS2-based RNA immunoprecipitation assay in 7402 cell transfected with MS2bs-circ0060467, MS2bs- circ0060467-mt, or MS2bs-Rluc. **p < 0.01.

CircRNAs can work as ceRNAs to modulate miRNAs, thereby liberating miRNA-targeted genes. Hence, we evaluated if circ0060467 can function as a miRNA sponge and projected the possible molecular targets of circ0060467 on CircBank (http://www.circbank.cn/). The analysis revealed that the putative binding site for miR-6085 resides within the sequence of circ0060467 (Figure 3A). qRT-PCR analysis demonstrated low expression levels of miR-6085 in HCC cells (Figure 3B). Moreover, co-transfection with miR-6085 mimics led to a suppression of luciferase activity, an effect not observed in cells transfected with the mutant luciferase reporter (Figure 3C). Subsequently, knockdown of circ-0060467 in 7402 and 97h cell lines resulted in a significant increase in miR-6085 expression (Figure 3D). To validate the direct binding between circ0060467 and miR-6085, an MS2bp-MS2bs-based RNA immunoprecipitation (RIP) assay was conducted. MiR-6085 was highly enriched in the MS2bs-circ-0060467 group (Figure 3E), validating the direct associations between circ0060467 and miR-6085. Thus, circ0060467 effectively interacts with miR-6085, serving as a sponge for miR-6085 in the context of HCC.

### Circ0060467 regulates expression of GPX4 and AIFM in HCC

**Figure 4:**
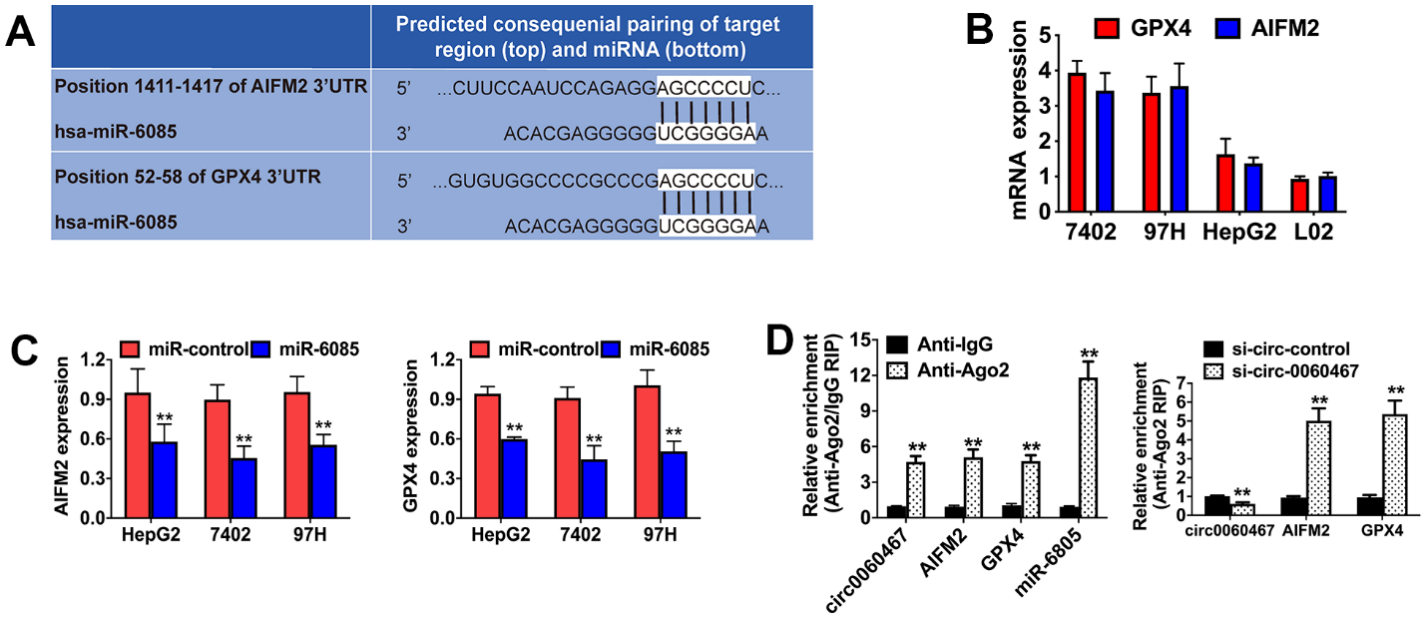
**Circ0060467 regulates the expression of GPX4 and AIFM in HCC.** (**A**) Predicted direct binding sites of hsa-miR-6085 (miR-6085) within the apoptosis inducing factor mitochondria associated 2 (AIFM2) and glutathione peroxidase 4 (GPX4) sequences. (**B**) Expression level of GPX4 and AIFM2 in the LO2 cell line and hepatocellular carcinoma cell lines. (**C**) Expression levels of GPX4 and AIFM2 after transfection with miR-6085 mimics. (**D**) RNA immunoprecipitation (RIP) assay detected the relative enrichment of circ0060467, GPX4, AIFM2, and miR-6085 in the anti-Argonaute 2 (Ago2) fraction (left); relative enrichment was detected by an Ago2-RIP assay (right).

The TargetScan algorithm was employed to investigate the possible downstream targets of miR-6085, revealing AIFM2 and GPX4 as putative target oncogenes (Figure 4A). To confirm the interactions of the predicted targets, expressions of AIFM2 and GPX4 were measured in HCC cell lines and were observed to be elevated (Figure 4B). In addition, miR-6085 mimics significantly inhibited AIFM2 and GPX4 levels in 7402, HepG2, and 97H cells, indicating that AIFM2 and GPX4 were both regulated by miR-6085 (Figure 4C). Then, Argonaute-2 (Ago2) RIP analyses were performed. It was found that circ0060467, AIFM2, GPX4, and miR-6085 were highly enriched in Ago2 complexes. Furthermore, suppression of circ0060467 enhanced AIFM2 and GPX4 enrichment in Ago2 fractions and largely inhibited circ0060467 enrichment in Ago2 complexes (Figure 4D), suggesting that circ0060467 competes with AIFM2 and GPX4 for binding to miR-6085.

### Circ0060467 suppression enhances HCC ferroptosis

**Figure 5:**
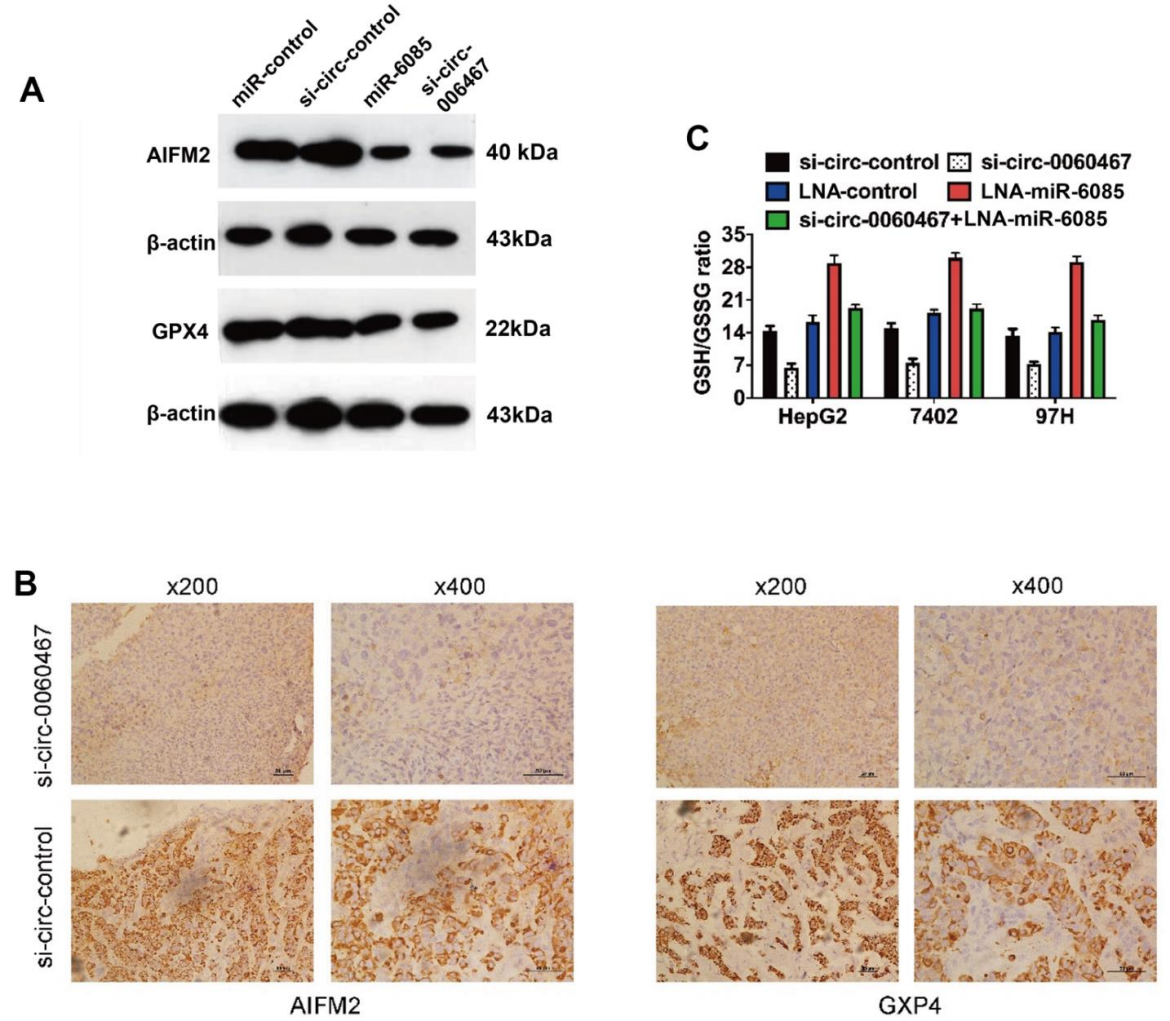
**Knockdown of circ0060467 promotes ferroptosis in HCC.** (**A**) Western blotting was performed to determine the expression levels of AIFM2, GPX4 and β-Actin. (**B**) Expression status of AIFM2 and GPX4 in hematoxylin-eosin-stained sections of harvested xenograft tumors. (**C**) Glutathione (GSH)/oxidized GSH (GSSG) ratio was determined with a GSH/GSSG Quantification Kit. **p < 0.01.

Interestingly, AIFM2 and GPX4 have been determined as vital regulators of tumor cell ferroptosis [22]. Consequently, suppression of circ0060467 enhances cell ferroptosis via the circ0060467/miR-6085/AIFM2 and GPX4 axis. Knockdown of circ0060467 or overexpression of miR-6085, AIFM2, and GPX4 was effective in suppressing 7402, HepG2, and 97H cells (Figure 5A). Additionally, immunohistochemical staining sections of harvested xenograft tumors validated that circ0060467 suppression apparently suppressed the levels of AIFM2 and GPX4 (Figure 5B). Moreover, circ0060467 knockdown led to a decrease in the GSH/GSSG ratio and mitigated GPX4 activation (Figure 5C).

## DISCUSSION

Substantial circRNAs were identified through high-throughput sequencing techniques and bioinformatics analyses. However, the significance of circRNAs in HCC has yet to be fully established [33]. In this study, we identified the levels of circRNAs in HCC, with circ0060467 found to be elevated in both HCC tissues and cell lines. Knockdown of circ0060467 suppressed HCC cell proliferation. These results show that circ0060467 plays a pivotal role in HCC progression, suggesting its potential as a novel diagnostic marker and treatment target for HCC. Nevertheless, due to the small sample size in this study, further investigation and validation in a larger independent cohort are warranted to explore the expression profile and clinicopathological significance of circ0060467 in HCC.

Recent studies have reported that ceRNAs influence the activity of miRNAs by acting as sponges to control gene expression in tumors [19, 34]. The relationship between circ0060467 and miR-6805 was validated through a series of functional assays. In this study, miR-6805 was shown to interact with circ0060467, AIFM2, and GPX4, suggesting that circ0060467 may act as a miR-6805 sponge to modulate AIFM2 and GPX4 expression via a ceRNA mechanism. Circ0060467 functions as a ceRNA, sponging miR-6805 for AIFM2 and GPX4 in HCC. Initially, bioinformatic analyses suggested that circ0060467 and the 3’-untranslated region (UTR) of AIFM2 and GPX4 contain specific binding sites for miR-6805. This prediction was verified through luciferase reporter and MS2bp-MS2bs-based RIP assays. Furthermore, AIFM2 and GPX4 expression were reduced following circ0060467 knockdown. Finally, the effects of circ0060467 knockdown were reversed by suppressing miR-6805 expression. Similar to circRNAs in the liver metastasis of breast cancer [35, 36], these findings demonstrate that circ0060467 regulates AIFM2 and GPX4 expression by sponging miR-6805 via a ceRNA mechanism.

Ferroptosis constitutes a form of necrotic cell demise facilitated by the oxidative alteration of phospholipid membranes through an iron-dependent mechanism [37, 38]. A pivotal aspect of this biological process involves cysteine depletion, leading to the depletion of the intracellular glutathione (reduced) (GSH) reservoir, ultimately triggering cellular apoptosis [22]. Initially, the regulation of ferroptosis was attributed to GPX4, an enzyme responsible for reducing phospholipid hydroperoxides [39, 40] and radical-trapping antioxidants [41, 42]. But recent study implies that flavoprotein AIFM2 acts as an antiferroptotic gene. AIFM2, also identified as ferroptosis suppressor protein 1 (FSP1), provides protection against ferroptosis induced by GPX4 deletion [43]. In this study, the downregulation of circ0060467 or the overexpression of miR-6805 was observed to suppress AIFM2 and GPX4 expression in HCC cell lines and xenograft tumors. Additionally, the knockdown of circ0060467 resulted in a diminished GSH/GSSG ratio in HCC cell lines. Therefore, our findings support the conclusion that the suppression of circ0060467 enhances ferroptosis in HCC.

## CONCLUSIONS

In conclusion, circ0060467 is highly expressed in HCC, and its depletion resulted in the suppression of HCC cell proliferation. Functioning as a ceRNA, circ0060467 played a pivotal role in the regulation of AIFM2 and GPX4 levels by sponging miR-6805. The circ0060467/miR-6805/AIFM2 and circ0060467/miR-6805/GPX4 axes were identified as key mediators in HCC progression through the modulation of ferroptosis. Consequently, circ0060467 is a potential novel diagnostic marker and a potential effective target to therapy for HCC. To facilitate the clinical application of circ0060467 as a targetable molecule in future treatments, it is imperative to conduct further investigations involving a larger cohort of samples.

## MATERIALS AND METHODS

### Cell lines and culture

The 97H and BEL-7402 cell lines were procured from the American Type Culture Collection (ATCC, Manassas, VA, USA). These cell lines were cultured in DMEM (Invitrogen, USA) supplemented with 10% fetal bovine serum (Gibco, USA) under conditions of 5% CO_2_ at 37° C. After resuscitating from frozen aliquots, cells were strictly passaged in less than 6 months and were verified by DNA fingerprinting every 6 months. The Ethical Committee of the First Affiliated Hospital of University of South China permitted this study (approval no. 2021110714001). The study strictly adhered to the principles outlined in the Declaration of Helsinki (revised in 2013). Before the commencement of the study, informed consent was obtained from all participating patients.

### qRT-PCR assay

TRIzol reagent (Life Technologies, Carlsbad, CA, USA) was utilized to extract total RNA from cells and tissues. The PrimeScript RT reagent kit (Takara Bio, Dalian, China) was used for cDNA synthesis, and qRT-PCR was carried out using SYBR Premix Ex Taq (Takara Bio) on a CFX96 Real-time PCR system (Bio-Rad, Hercules, CA, USA). To assess the abundances of target transcripts, U6, along with β-actin (used as housekeeping genes), served as controls. Relative fold changes in expressions were calculated using the 2^−ΔΔCt^ method.

### RNase R digestion assay

RNA isolated from 97H and 7402 cells were treated using RNase R (Epicenter Technologies, Madison, WI, USA) or a buffer as control. In the RNase R group, total RNA (2 μg) was mixed with RNase R (3 U/μg) and incubated for 20 minutes at 37° C. The β-Actin was used as an internal control.

### Actinomycin D assay

Cells (at a density of 1×10^5^) were seeded in 6-well plates and exposed to actinomycin D (2 mg/L; Sigma, USA). The treated cells were harvested at 8, 16, and 24 h intervals. The circ0060467 and MYBL2 mRNA levels were determined by qRT-PCR test.

### Cell counting kit-8 (CCK-8) and Transwell assays

A CCK-8 assay (Dojindo Laboratories, Japan, Cat. # ck04) was performed to evaluate cell proliferation. Cells (1×10^3^ cells/well) were cultured in 96-well plates and thereafter transfected at 48 h prior to the addition of the CCK-8 solution (10 μL) to each well. After 2 h of incubation at 37° C, light absorbance at 450 nM was detected in a microtiter plate reader (Epoch2; BioTek, Winooski, VT, USA). With regards to the Transwell assay, cells (1×10^4^ cells/mL) were inoculated into a migration chamber (BD Biosciences, Franklin Lakes, NJ, USA), after which the medium (supplemented with 10% FBS) was supplemented to the lower chamber as an attractant. At 24 h post-incubation, the cells were fixed with methanol, stained with crystal violet (0.1%), and microscopically counted. Assays were performed in triplicate.

### Mouse xenograft models

The procedures used for the animal assays were permitted by the Institutional Animal Experimental Ethical Committee of the First Affiliated Hospital of the University of South China (approval no. 2021110714001). Female BALB/c nude mice (4-week-old; three mice per group) were subcutaneously injected with 2×10^6^ BEL-7402 cells. Intratumoral administration (40 μL of si-circ0060467 or negative control siRNA) was performed every 4 days and tumor volumes were assessed. Then, the mice were properly sacrificed and tumor immunohistochemistry was performed.

### Immunohistochemistry

Section slides with tissue were deparaffinized in xylene and infiltrated with graded concentrations of ethanol (100%, 95%, 85%, and 75%). After performing the blocking of endogenous peroxidase activity and antigen retrieval, the Ki-67 (Proteintech, China, 1:200) antibody was added and incubated overnight at 4° C. The sections were stained with a diaminobenzidine (DAB) substrate (Dako) after 20 minutes of incubation at room temperature. The sections were stained with hematoxylin after DAB treatment. The sections slides were dried, and the degree of staining and the positive range were measured under a microscope. The degree of staining (0-3 points) was negative, light yellow, light brown, and dark brown; the positive range (1-4 points) was respectively 0-25%, 26-50%, 51-75%, 76-100%, and the final scores were added for comparison. And we have added the related content to the Methods section according to the degree of staining and the positive range under a light microscope.

### Luciferase reporter assay

Luciferase reporter vectors with full lengths 3’-UTR of AIFM2 and GPX4 or circ0060467 were established. Then, mutant luciferase reporter vectors were generated using the QuikChange XL Site-directed Mutagenesis Kit (Stratagene, La Jolla, CA, USA). BEL-7402 cells were seeded into 96-well plates and co-transfected with luciferase reporter vectors and miR-6805 mimics or miR-6805 locked nucleic acids (LNA). Cells were incubated for 48 h, after which luciferase activity was quantified using dual-luciferase reporter assays (Promega, Madison, WI, USA). Cells (density, 5×10^3^) were co-transfected with the established vectors and miR-6805 mimics. After incubation for 48 h, a dual-luciferase reporter assay system (Promega, WI, USA) was used to evaluate relative luciferase activity.

### RIP assays

BEL-7402 cells were separately transfected with MS2bs-circ0097009 mt, MS2bs-circ0097009, or the blank control MS2bs-Rluc, with MS2bp-GFP using Lipofectamine 2000 (Invitrogen, Carlsbad, CA, USA). After incubation for 48 h, the RIP assay was performed using the Magna RIP RNA-Binding Protein Immunoprecipitation Kit (Millipore, Burlington, MA, USA). The relative levels of miR-6805 were quantified after purification of the RNA complexes. The anti-Ago2-RIP assay was done using anti-Ago2 antibodies (Millipore, Burlington, MA, USA). The abundances of circ0060467, AIFM2 and GPX4, and miR-6805 were accurately quantified after RNA purification.

### Western blot analysis

After extraction, total proteins were separated by 10% SDS-PAGE and transferred onto PVDF membranes (Millipore, Burlington, MA, USA), which were blocked at room temperature using 5% skim milk for 1 h. The membrane was then incubated with primary anti-AIFM2 (1:1,000; Affinity Biosciences, Zhenjiang, China, Cat. #: DF8636) or anti-GPX4 (1:1,000; Abcam, Cambridge, MA, USA, Cat. # ab125066) or anti-β-Actin (1:1,000; Abcam, Cat. # ab8245) antibody. Secondary antibodies (1:5,000; Cell Signaling Technology, Danvers, MA, USA, Cat. # 7074) were used. Chemiluminescence was used for the detection.

### Evaluation of glutathione (GSH)/oxidized GSH (GSSG) ratios

The GSH/GSSG was determined using the GSH/GSSG Quantification Kit II (Dojindo, Shanghai, China). Preparation of the sample and standard solutions, as well as the evaluation of concentrations, were strictly conducted according to the manufacturer’s protocols. A microplate reader (FlexStation 3; Molecular Devices, San Jose, CA, USA) was used to calculate relative levels.

### Statistical analyses

The SPSS 25.0 (IBM, SPSS, Chicago, IL, USA) for Windows was used for data analyses. Data were shown as mean ± SEM. Among-group comparisons were performed by one-way ANOVA while between-group comparisons were done by two-tailed t-tests. *p* < 0.05 denoted significance.

### Data availability

Authors can provide all of the datasets on reasonable request.

## References

[1] Sung H, Ferlay J, Siegel RL, Laversanne M, Soerjomataram I, Jemal A, Bray F. Global Cancer Statistics 2020: GLOBOCAN Estimates of Incidence and Mortality Worldwide for 36 Cancers in 185 Countries. CA Cancer J Clin. 2021; 71:209–49. 10.3322/caac.21660 33538338

[2] Bray F, Ferlay J, Soerjomataram I, Siegel RL, Torre LA, Jemal A. Global cancer statistics 2018: GLOBOCAN estimates of incidence and mortality worldwide for 36 cancers in 185 countries. CA Cancer J Clin. 2018; 68:394–424. 10.3322/caac.21492 30207593

[3] Nordenstedt H, White DL, El-Serag HB. The changing pattern of epidemiology in hepatocellular carcinoma. Dig Liver Dis. 2010 (Suppl 3); 42:S206–14. 10.1016/S1590-8658(10)60507-5 20547305 PMC3392755

[4] Schlabe S, Rockstroh JK. Advances in the treatment of HIV/HCV coinfection in adults. Expert Opin Pharmacother. 2018; 19:49–64. 10.1080/14656566.2017.1419185 29252031

[5] Villanueva A. Hepatocellular Carcinoma. N Engl J Med. 2019; 380:1450–62. 10.1056/NEJMra1713263 30970190

[6] Vitale A, Trevisani F, Farinati F, Cillo U. Treatment of Hepatocellular Carcinoma in the Precision Medicine Era: From Treatment Stage Migration to Therapeutic Hierarchy. Hepatology. 2020; 72:2206–18. 10.1002/hep.31187 32064645

[7] Hou J, Zhang H, Sun B, Karin M. The immunobiology of hepatocellular carcinoma in humans and mice: Basic concepts and therapeutic implications. J Hepatol. 2020; 72:167–82. 10.1016/j.jhep.2019.08.014 31449859

[8] Craig AJ, von Felden J, Garcia-Lezana T, Sarcognato S, Villanueva A. Tumour evolution in hepatocellular carcinoma. Nat Rev Gastroenterol Hepatol. 2020; 17:139–52. 10.1038/s41575-019-0229-4 31792430

[9] Topper MJ, Vaz M, Marrone KA, Brahmer JR, Baylin SB. The emerging role of epigenetic therapeutics in immuno-oncology. Nat Rev Clin Oncol. 2020; 17:75–90. 10.1038/s41571-019-0266-5 31548600 PMC7254932

[10] Kudo M. A Paradigm Change in the Treatment Strategy for Hepatocellular Carcinoma. Liver Cancer. 2020; 9:367–77. 10.1159/000507934 32999864 PMC7506281

[11] Kloeckner R, Galle PR, Bruix J. Local and Regional Therapies for Hepatocellular Carcinoma. Hepatology. 2021; 73:137–49. 10.1002/hep.31424 32557715

[12] Vo JN, Cieslik M, Zhang Y, Shukla S, Xiao L, Zhang Y, Wu YM, Dhanasekaran SM, Engelke CG, Cao X, Robinson DR, Nesvizhskii AI, Chinnaiyan AM. The Landscape of Circular RNA in Cancer. Cell. 2019; 176:869–81.e13. 10.1016/j.cell.2018.12.021 30735636 PMC6601354

[13] Kristensen LS, Andersen MS, Stagsted LVW, Ebbesen KK, Hansen TB, Kjems J. The biogenesis, biology and characterization of circular RNAs. Nat Rev Genet. 2019; 20:675–91. 10.1038/s41576-019-0158-7 31395983

[14] Yu J, Xu QG, Wang ZG, Yang Y, Zhang L, Ma JZ, Sun SH, Yang F, Zhou WP. Circular RNA cSMARCA5 inhibits growth and metastasis in hepatocellular carcinoma. J Hepatol. 2018; 68:1214–27. 10.1016/j.jhep.2018.01.012 29378234

[15] Zhang X, Wang S, Wang H, Cao J, Huang X, Chen Z, Xu P, Sun G, Xu J, Lv J, Xu Z. Circular RNA circNRIP1 acts as a microRNA-149-5p sponge to promote gastric cancer progression via the AKT1/mTOR pathway. Mol Cancer. 2019; 18:20. 10.1186/s12943-018-0935-5 30717751 PMC6360801

[16] Hoshida Y, Fuchs BC, Tanabe KK. Prevention of hepatocellular carcinoma: potential targets, experimental models, and clinical challenges. Curr Cancer Drug Targets. 2012; 12:1129–59. 10.2174/15680096112091129 22873223 PMC3776581

[17] Quinn JJ, Chang HY. Unique features of long non-coding RNA biogenesis and function. Nat Rev Genet. 2016; 17:47–62. 10.1038/nrg.2015.10 26666209

[18] Wang J, Li H, Liang Z. circ-MYBL2 Serves As A Sponge For miR-361-3p Promoting Cervical Cancer Cells Proliferation And Invasion. Onco Targets Ther. 2019; 12:9957–64. 10.2147/OTT.S218976 31819492 PMC6877451

[19] Wei Y, Chen X, Liang C, Ling Y, Yang X, Ye X, Zhang H, Yang P, Cui X, Ren Y, Xin X, Li H, Wang R, et al. A Noncoding Regulatory RNAs Network Driven by Circ-CDYL Acts Specifically in the Early Stages Hepatocellular Carcinoma. Hepatology. 2020; 71:130–47. 10.1002/hep.30795 31148183

[20] Qi SX, Sun H, Liu H, Yu J, Jiang ZY, Yan P. Role and mechanism of circ-PRKCI in hepatocellular carcinoma. World J Gastroenterol. 2019; 25:1964–74. 10.3748/wjg.v25.i16.1964 31086464 PMC6487381

[21] Xie B, Zhao Z, Liu Q, Wang X, Ma Z, Li H. CircRNA has_circ_0078710 acts as the sponge of microRNA-31 involved in hepatocellular carcinoma progression. Gene. 2019; 683:253–61. 10.1016/j.gene.2018.10.043 30342168

[22] Dixon SJ, Lemberg KM, Lamprecht MR, Skouta R, Zaitsev EM, Gleason CE, Patel DN, Bauer AJ, Cantley AM, Yang WS, Morrison B 3rd, Stockwell BR. Ferroptosis: an iron-dependent form of nonapoptotic cell death. Cell. 2012; 149:1060–72. 10.1016/j.cell.2012.03.042 22632970 PMC3367386

[23] Stockwell BR, Friedmann Angeli JP, Bayir H, Bush AI, Conrad M, Dixon SJ, Fulda S, Gascón S, Hatzios SK, Kagan VE, Noel K, Jiang X, Linkermann A, et al. Ferroptosis: A Regulated Cell Death Nexus Linking Metabolism, Redox Biology, and Disease. Cell. 2017; 171:273–85. 10.1016/j.cell.2017.09.021 28985560 PMC5685180

[24] Hassannia B, Vandenabeele P, Vanden Berghe T. Targeting Ferroptosis to Iron Out Cancer. Cancer Cell. 2019; 35:830–49. 10.1016/j.ccell.2019.04.002 31105042

[25] Yi J, Zhu J, Wu J, Thompson CB, Jiang X. Oncogenic activation of PI3K-AKT-mTOR signaling suppresses ferroptosis via SREBP-mediated lipogenesis. Proc Natl Acad Sci USA. 2020; 117:31189–97. 10.1073/pnas.2017152117 33229547 PMC7733797

[26] Ubellacker JM, Tasdogan A, Ramesh V, Shen B, Mitchell EC, Martin-Sandoval MS, Gu Z, McCormick ML, Durham AB, Spitz DR, Zhao Z, Mathews TP, Morrison SJ. Lymph protects metastasizing melanoma cells from ferroptosis. Nature. 2020; 585:113–8. 10.1038/s41586-020-2623-z 32814895 PMC7484468

[27] Friedmann Angeli JP, Krysko DV, Conrad M. Ferroptosis at the crossroads of cancer-acquired drug resistance and immune evasion. Nat Rev Cancer. 2019; 19:405–14. 10.1038/s41568-019-0149-1 31101865

[28] Wu J, Minikes AM, Gao M, Bian H, Li Y, Stockwell BR, Chen ZN, Jiang X. Intercellular interaction dictates cancer cell ferroptosis via NF2-YAP signalling. Nature. 2019; 572:402–6. 10.1038/s41586-019-1426-6 31341276 PMC6697195

[29] Zou Y, Henry WS, Ricq EL, Graham ET, Phadnis VV, Maretich P, Paradkar S, Boehnke N, Deik AA, Reinhardt F, Eaton JK, Ferguson B, Wang W, et al. Plasticity of ether lipids promotes ferroptosis susceptibility and evasion. Nature. 2020; 585:603–8. 10.1038/s41586-020-2732-8 32939090 PMC8051864

[30] Zou Y, Palte MJ, Deik AA, Li H, Eaton JK, Wang W, Tseng YY, Deasy R, Kost-Alimova M, Dančík V, Leshchiner ES, Viswanathan VS, Signoretti S, et al. A GPX4-dependent cancer cell state underlies the clear-cell morphology and confers sensitivity to ferroptosis. Nat Commun. 2019; 10:1617. 10.1038/s41467-019-09277-9 30962421 PMC6453886

[31] Dong M, Li P, Xie Y, Wang Z, Wang R. CircMYBL2 regulates the resistance of cervical cancer cells to paclitaxel via miR-665-dependent regulation of EGFR. Drug Dev Res. 2021; 82:1193–205. 10.1002/ddr.21834 34046939

[32] Sun YM, Wang WT, Zeng ZC, Chen TQ, Han C, Pan Q, Huang W, Fang K, Sun LY, Zhou YF, Luo XQ, Luo C, Du X, Chen YQ. circMYBL2, a circRNA from MYBL2, regulates FLT3 translation by recruiting PTBP1 to promote FLT3-ITD AML progression. Blood. 2019; 134:1533–46. 10.1182/blood.2019000802 31387917 PMC6839953

[33] Wu S, Turner KM, Nguyen N, Raviram R, Erb M, Santini J, Luebeck J, Rajkumar U, Diao Y, Li B, Zhang W, Jameson N, Corces MR, et al. Circular ecDNA promotes accessible chromatin and high oncogene expression. Nature. 2019; 575:699–703. 10.1038/s41586-019-1763-5 31748743 PMC7094777

[34] Dragomir MP, Kopetz S, Ajani JA, Calin GA. Non-coding RNAs in GI cancers: from cancer hallmarks to clinical utility. Gut. 2020; 69:748–63. 10.1136/gutjnl-2019-318279 32034004

[35] Zeng Y, Du W, Huang Z, Wu S, Ou X, Zhang J, Peng C, Sun X, Tang H. Hsa_circ_0060467 promotes breast cancer liver metastasis by complexing with eIF4A3 and sponging miR-1205. Cell Death Discov. 2023; 9:153. 10.1038/s41420-023-01448-4 37160894 PMC10169853

[36] Liu P, Wang Z, Ou X, Wu P, Zhang Y, Wu S, Xiao X, Li Y, Ye F, Tang H. The FUS/circEZH2/KLF5/ feedback loop contributes to CXCR4-induced liver metastasis of breast cancer by enhancing epithelial-mesenchymal transition. Mol Cancer. 2022; 21:198. 10.1186/s12943-022-01653-2 36224562 PMC9555172

[37] Conrad M, Angeli JP, Vandenabeele P, Stockwell BR. Regulated necrosis: disease relevance and therapeutic opportunities. Nat Rev Drug Discov. 2016; 15:348–66. 10.1038/nrd.2015.6 26775689 PMC6531857

[38] Lyu N, Zeng Y, Kong Y, Chen Q, Deng H, Ou S, Bai Y, Tang H, Wang X, Zhao M. Ferroptosis is involved in the progression of hepatocellular carcinoma through the circ0097009/miR-1261/SLC7A11 axis. Ann Transl Med. 2021; 9:675. 10.21037/atm-21-997 33987373 PMC8106082

[39] Yang WS, SriRamaratnam R, Welsch ME, Shimada K, Skouta R, Viswanathan VS, Cheah JH, Clemons PA, Shamji AF, Clish CB, Brown LM, Girotti AW, Cornish VW, et al. Regulation of ferroptotic cancer cell death by GPX4. Cell. 2014; 156:317–31. 10.1016/j.cell.2013.12.010 24439385 PMC4076414

[40] Friedmann Angeli JP, Schneider M, Proneth B, Tyurina YY, Tyurin VA, Hammond VJ, Herbach N, Aichler M, Walch A, Eggenhofer E, Basavarajappa D, Rådmark O, Kobayashi S, et al. Inactivation of the ferroptosis regulator Gpx4 triggers acute renal failure in mice. Nat Cell Biol. 2014; 16:1180–91. 10.1038/ncb3064 25402683 PMC4894846

[41] Zilka O, Shah R, Li B, Friedmann Angeli JP, Griesser M, Conrad M, Pratt DA. On the Mechanism of Cytoprotection by Ferrostatin-1 and Liproxstatin-1 and the Role of Lipid Peroxidation in Ferroptotic Cell Death. ACS Cent Sci. 2017; 3:232–43. 10.1021/acscentsci.7b00028 28386601 PMC5364454

[42] Shah R, Shchepinov MS, Pratt DA. Resolving the Role of Lipoxygenases in the Initiation and Execution of Ferroptosis. ACS Cent Sci. 2018; 4:387–96. 10.1021/acscentsci.7b00589 29632885 PMC5879472

[43] Doll S, Freitas FP, Shah R, Aldrovandi M, da Silva MC, Ingold I, Goya Grocin A, Xavier da Silva TN, Panzilius E, Scheel CH, Mourão A, Buday K, Sato M, et al. FSP1 is a glutathione-independent ferroptosis suppressor. Nature. 2019; 575:693–8. 10.1038/s41586-019-1707-0 31634899

